# Psychiatric readmission rates in a multi-level mental health care system – a descriptive population cohort study

**DOI:** 10.1186/s12913-021-06391-7

**Published:** 2021-04-23

**Authors:** Eva Lassemo, Lars Henrik Myklebust, Damiano Salazzari, Jorid Kalseth

**Affiliations:** 1 SINTEF Digital, Health Research, P.O. Box 4760 Torgarden, NO-7465 Trondheim, Norway; 2 Sykehuset Innlandet HF, P.O. Box 4453, 2326 Hamar, Norway; 3 Department of Neurosciences, Biomedicine and Movement Sciences, University of Verona, Piazzale L.A. Scuro, 10, 37134 Verona, Italy

**Keywords:** Readmission, Psychiatry, Multi-level mental health care system, Survival analysis

## Abstract

**Background:**

Readmission rates are frequently used as a quality indicator for health care, yet their validity for evaluating quality is unclear. Published research on variables affecting readmission to psychiatric hospitals have been inconsistent. The Norwegian specialist mental health care system is characterized by a multi-level structure; hospitals providing specialized -largely unplanned care and district psychiatric centers (DPCs) providing generalized -more often planned care. In certain service systems, readmission may be an integral part of individual patients’ treatment plan.

The aim of the present study was to describe and examine the task division in a multi-level health care system. This we did through describing differences in patient population (age, sex, diagnosis, substance abuse comorbidity and length of stay) and admissions types (unplanned vs. planned) treated at different levels (hospital, DPC or both), and by examining whether readmission risk differ according to type and place of treatment of index-admission and travel-time to nearest hospital and DPC.

**Methods:**

In this population-based cohort study using administrative data we included all individuals aged 18 and older who were discharged from psychiatric inpatient care with an ICD-10 diagnosis F2-F6 (“functional mental disorders”) in 2012. Selecting each individual’s first discharge during 2012 as index gave *N* = 16,185 for analyses following exclusions. Analysis of readmission risk were done using Kaplan-Maier failure curves.

**Results:**

Overall, 15.1 and 47.7% of patients were readmitted within 30 and 365 days, respectively. Unplanned admission patients were more likely to be readmitted within 30 days than planned patients. Those transferred between hospital and DPC during index admission were more likely to be readmitted within 365 days, and to experience planned readmission. Patients with short travel time were more likely to have unplanned readmission, while patients with long travel time were more likely to have planned readmission.

**Conclusions:**

DPCs and hospitals fill different purposes in the Norwegian health care system, which is reflected in different patient populations. Differences in short term readmission rates between hospitals and DPCs disappeared when type of admission (unplanned/planned) was considered. The results stress the importance of addressing differences in organisation and task distribution when comparing readmission rates between mental health systems.

## Background

Deinstitutionalization has been the general trend in psychiatric health care services since the mid-1900s This restructuring has been from primarily hospital-based services towards extramural and outreach services consisting of community mental health teams, crisis teams and assertive outreach teams. A consequence of the deinstitutionalization has been the arise of “revolving door” patients [1]. This has led to an increased focus on readmission rates as quality indicators. Readmission rates are used as a quality indicator for inpatient health care [2, 3], yet their validity for evaluating quality is unclear [4].

A limited number of variables are consistently associated with readmission [4, 5], with time since discharge and number of previous admissions being the prominent ones. Reviewing the literature, Tulloch et al. [6] identify diagnosis, gender and age as individual level variables associated with readmission, albeit with little consistency in the size and significance of the effect, or its direction. Systematic literature reviews on the association of pre-discharge factors [7], post-discharge factors [8], and physical comorbidity [9] with risk of psychiatric readmission reveal a vast variety of possible factors with low levels of consistency. The most consistently significant predictor of readmission also in these reviews was previous hospitalizations.

Readmission rates also varies between countries [10]. However, knowledge regarding how health system characteristics affect readmission rates is scarce [11]. Among factors that previously have been identified as to impact readmission rates are continuity of care and access to aftercare post discharge [8, 12]. Contrary, Sytema and Burgess [13] found that consumption of community-based care had no effect on the relative risk of readmission. Neighborhood effects, in terms of e.g. deprived socioeconomic status [14] or geographic clustering [15], have been found to impact utilization of psychiatric health care services.

Norway is an interesting case in this respect. A special feature of the mental health care system in Norway is that it has both a large number of psychiatric hospital beds per capita and highly developed community mental health services [16]. Furthermore, Norway has a, compared to other European countries, high rehospitalization rate both at 30- and 365 days post discharge [10].

In studying the de- and trans-institutionalization of psychiatric health care in Norway, Pedersen and Kolstad [17] identified six distinct periods in the years 1950–2007. Starting in 1985, district psychiatric centers (DPCs) gradually replaced psychiatric nursing homes (PNHs) [18] and have developed into filling the function of decentralized mental hospitals or community mental health centers with inpatient wards. While patients had PNHs as their permanent residence, the DPCs are intended to provide short-term inpatient care, daycare and outpatient services for the local community. The mental health care system in Norway can be characterized as a multi-level system, with hospitals providing specialized (largely unplanned) care, and DPCs providing generalized (more often planned or elective) care. About 40% of the inpatient bed capacity is within DPCs and the major part of outpatient treatment, nearly 90%, take place at the DPCs [19]. Moreover, a large portion of mental health care is provided by general physicians (GPs) and municipal mental health workers in the primary health services [20].

The inpatient bed capacity continues to decline in Norway. There has been a steady decline and a halving of bed rates per population aged 18 years or more the last two decades [19], and the rates for DPCs also started to decline from 2005. With inpatient beds being removed without being replaced by adequate alternatives in primary care, the rate of unplanned admissions may rise [21]. Additionally, Ose et al. [20] found that many patients in specialist mental health services need municipal health- and social services. For certain patient groups, hospital admission may play an important role in the health care provided. Thus, not all readmissions are unwanted or to be interpreted as representing poor quality. Pedersen & Kolstad [17] found a general trend in fewer beds but not fewer patients treated. They found that an increase in number of discharges was facilitated by a reduction of average length of stay (LOS). The discharge rate has been relatively stable also in recent years compared to the decrease in the bed rate [19].

**Fig. 1:**
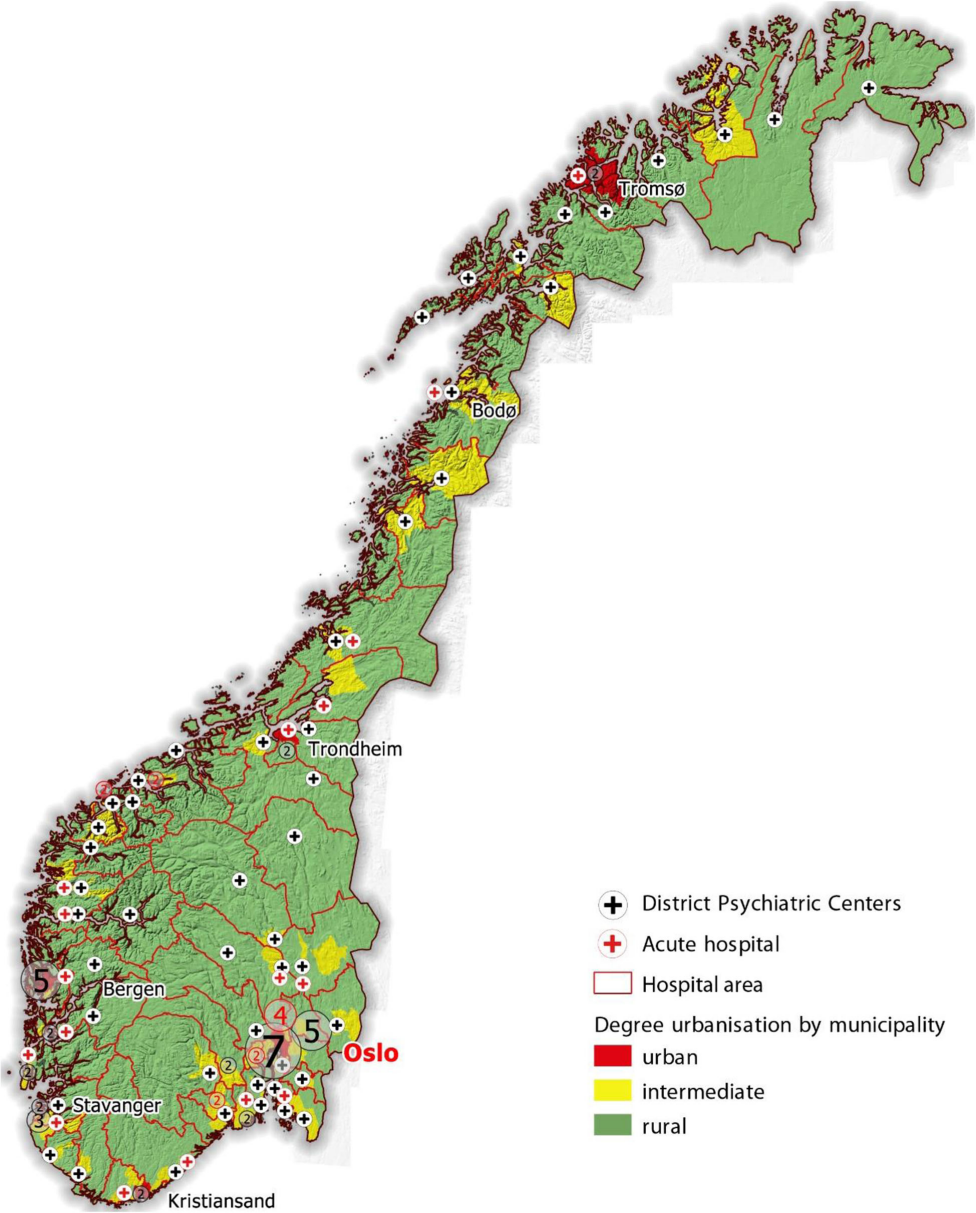
Map showing distribution of psychiatric hospital wards and district psychiatric centers in Norway. The map is the researchers’ own work

As can be seen in Fig. 1, hospitals with psychiatric wards and DPCs are dispersed over much of the populated areas of Norway. Still, distance to nearest facility is long for a sizable share of the population. Myklebust et al. [22, 23] found that geographical distance to psychiatric beds had surprisingly small effect on utilization. The deinstitutionalization has on the other hand been found to affect rates of acute-admissions and involuntary treatment of psychiatric patients [24, 25].

As Durbin et al. [4] states, changes in the organization and set-up of psychiatric health care renders early research on the topic less useful and relevant. Recent research has focused on identification of individual risk factors and care planning related to readmission risk, e.g. [26, 27]. The apparent dearth of publications with a systems stance from the later years is somewhat astonishing [11].System level variables such as distribution of tasks or travel time/geography has thus far received little attention in the readmission literature.

Therefore, the aims of the present study were 1) to describe and examine readmission risk within 1 year from psychiatric index admission to hospitals and DPCs in a multi-level mental health care system, 2) to examine whether readmission risk differ according to type (unplanned vs planned) and place (hospital vs DPC) of treatment of index admission, and 3) to examine effects of travel-time to nearest hospital and DPC on readmission risk.

## Methods

### Study design and setting

This study is a population-based cohort study using health administrative data to study readmission risk within 1 year from psychiatric index admissions to hospitals and DPCs in Norway, as well as differences in readmission risk dependent on whether index admission and readmission was planned or unplanned, and to examine effects of travel-time to nearest hospital and DPC on readmission risk.

The Norwegian health care system is universal and publicly funded, with exception of a deductible for outpatient- and GP services (2012 annual maximum $240). Four regional health authorities overseen by the Ministry of Health and Care Services are responsible for specialist health services for the population. Specialist psychiatric health care is provided through in total 19 hospital trusts. Each hospital trust in turn is comprised of hospital departments and a varying number of DPCs, 73 in total. Hospital departments includes both geographically standalone psychiatric hospital and psychiatric wards at general hospitals, however, all are organizationally integrated with other specialist health services within the 19 hospital trusts. The location of the hospital departments and DPCs can be seen in Fig. 1. The mental health care system is organized by geography. The catchment areas of each hospital trust encompasses one or more DPCs, while the catchment area of a DPC cover one or more municipalities, which in turn are responsible for providing a range of different health and care services including primary care, mental health workers, housing, day activities etc. to their population. In some cases, such as some larger cities, the population of a municipality are divided to more than one DPC.

### Data and study population

Patient administrative records for specialist inpatient health services for the years 2012–2014 were obtained from the Norwegian Patient Register (NPR). The NPR is a national health register, owned and administered by the Norwegian Directorate of Health, containing information on all patients waiting for, or having received, treatment in publicly financed specialist health care services. The NPR contains information on the patients’ date of birth, sex and postal code (aggregated to municipality of residence before handed over to the project) in addition to all clinical and administrative information regarding the stay, such as dates in and out, diagnoses, place of treatment, unplanned/planned admission.

This project was part of the larger EU FP7-funded research project CEPHOS-LINK (Comparative Effectiveness research on Psychiatric HOSpitalisation by record LINKage of large administrative data sets) on psychiatric re-hospitalization in Europe [10] and followed the same study protocol for identifying the study population. Ethical approval was obtained from the Regional Committee for Medical and Health Research Ethics for central Norway, approval number 2015/852. Informed consent is not applicable for population data gathered without informed consent. We included all individuals aged 18 and older who were discharged from psychiatric health care services with a so-called “functional mental disorder”, covering schizophrenia, affective, anxiety and personality disorders with an ICD-10 [28] main diagnosis F2-F6 in 2012. In Norway forensic psychiatry is integrated in the psychiatric services, and thus exclusion of forensic units was not fully possible. We have so-called “secure wards” at both regional and county level, regional wards could be identified and hence excluded from the study. Because of the usually very low turnover of these patient groups potential errors should be very small. Selecting each individual’s first discharge during the year as index discharge gave *N* = 17,158. Exclusion criteria were missing data on the demographic variables sex, age or domicile, and incomplete data on index admission and/or readmission considering diagnosis or admission and discharge dates. In total 973 individuals were excluded, leaving *N* = 16,185 for analyses. All index admissions were followed for readmission up to 365 days post discharge.

As mentioned, all hospital admissions in NPR contain a range of data for each record, including date of admission and discharge. Coding consecutive admissions as one admission episode, length of stay (LOS) by episode was calculated. Type of admission was based on registration of degree of urgency of admission, where the code ‘without further ado/ waiting’ was coded as unplanned, and the code ‘planned or waiting more than 24 h’ was coded as planned. Place (hospital vs DPC) of admission was coded as mix if the episode of treatment consisted of (a) stay(s) in both hospital and DPC. The same variables were available regarding admissions and readmissions. Data on sex, age, ICD-10 diagnoses (main and secondary), and domicile were also collected from NPR.

Descriptive characteristics of the study population at index admission are examined. The characteristics examined are place of admission (hospital/DPC/mix), type of admission (unplanned/planned), sex, age, main diagnosis and travel time to nearest DPC and nearest hospital.

In order to calculate average travel time from place of residence for the Norwegian population to location of the DPC and hospital services, a Geographical Information System (GIS)-based method was applied. Three main types of data were used to evaluate the geographical distribution and the geographic access to any hospitals/DPC: the location of the hospitals/DPCs, the location of the population and road networks data. The location of all hospitals with psychiatric wards and DPCs were acquired during the mapping project of the CEPHOS-LINK project [29]. Secondly, for the whole country, population size and location data were obtained from GEOSTAT 1A [30], which is a European population grid dataset, representing Census data referring to the year 2011. This study used detailed and updated street networks data from HERE API [31], in order to accurately estimate travel time between hospitals/DPCs and population locations. The HERE API provides the information on the travel time taking into account the fastest routes using a car between one origin point and one or more destinations without optimizing the routes for current traffic conditions. The origin point used for all travel time calculations are the population centroid of the GEOSTAT 1A grid dataset, while the destinations are the hospitals and DPCs. For simplicity, the average minimum travel time (in minutes) between the population centroid to the nearest hospital and DPC was calculated for each municipality. This was based on an estimate of the potential location where most of the people lives.

### Analyses

All statistical analyses were performed using STATA version 15.1 [32]. Geographical analyses and maps were made using QGIS [33].

Survival analyses were used to graphically describe readmission rates at 30- and 365 days using Kaplan-Maier survival plots. Secondary, potentially statistically significant differences were analyzed. Unless otherwise stated, all presented results are statistically significant at the 95% level.

## Results

### Index admission individual characteristics

**Fig. 2:**
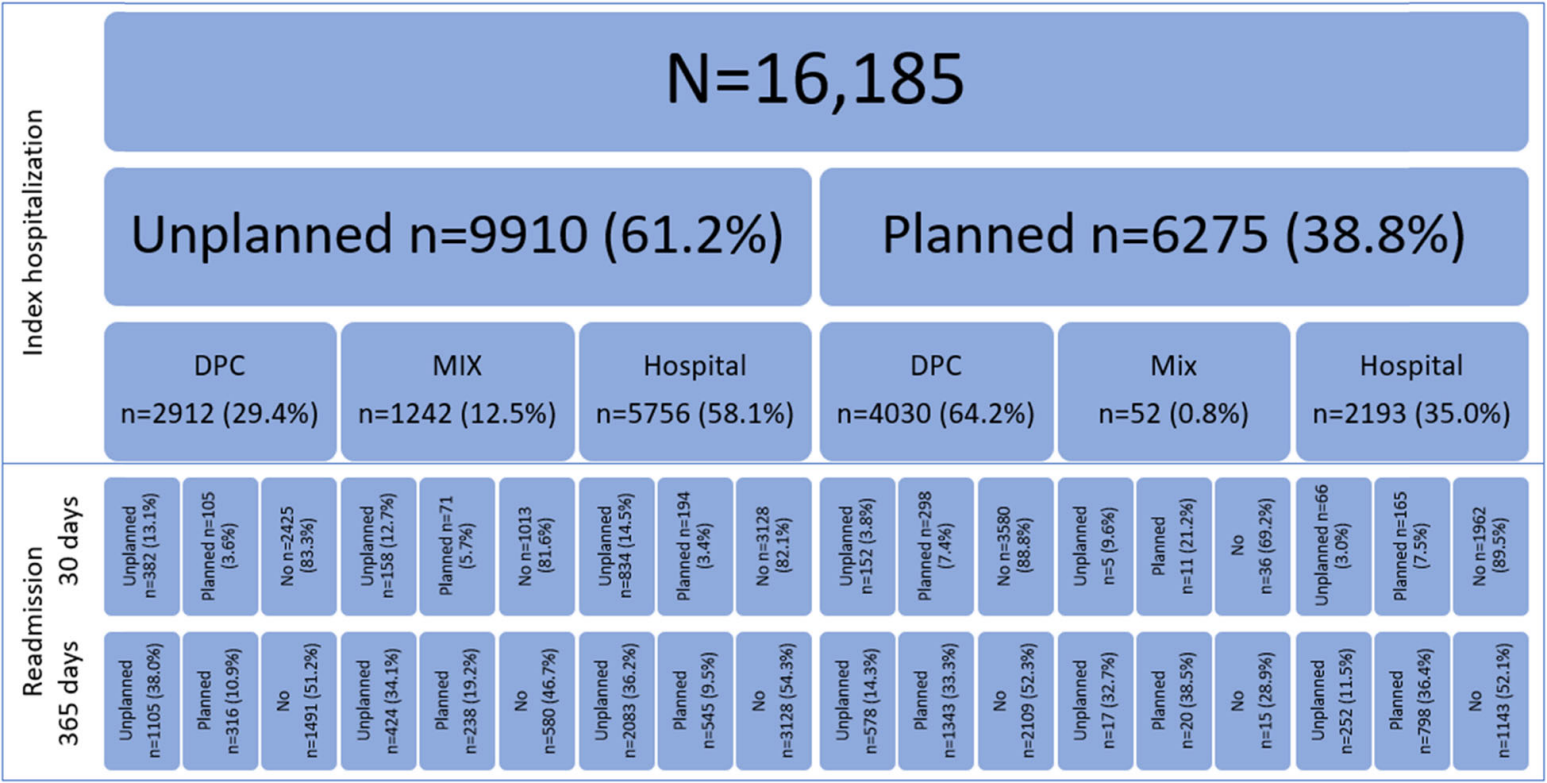
Study population, index admission and readmission, by unplanned/planned and place of index admission

**Fig. 3:**
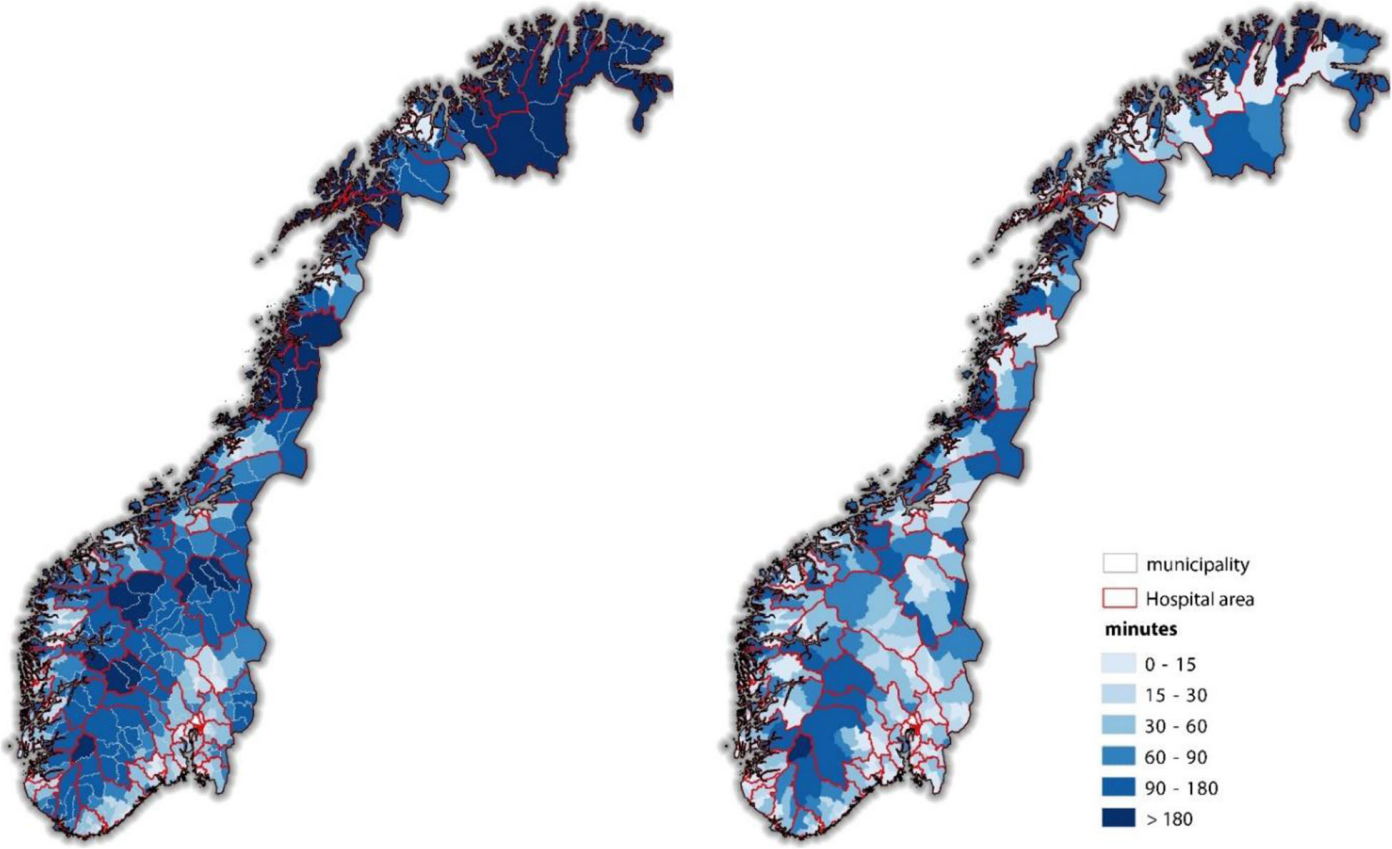
Maps showing left) average travel time at municipality level to nearest psychiatric hospital ward, and right) average travel time at municipality level to nearest DPC. The map is the researchers’ own work

Descriptive characteristics of the study population at index admission can be seen in Table 1 and Fig. 2. In the study population there were more women than men. The mean age was 43 (range 18–97, SD 16.4). The most common diagnosis was depression or other affective disorders (ICD-10 F32-F39), followed by schizophrenia or psychotic disorders (ICD-10 F2) and Neurotic disorders (ICD-10 F4). Mean and median LOS for the index episode was 34.8 and 14 days, respectively, and 30% had LOS < =7 days and 30% had LOS > 30 days. Overall, nearly half of the admissions were at a hospital only, and an additional 8 % were mixed stays involving both hospital and DPC. Of index admissions, near 39% were planned. Of unplanned index admissions, 58% were to hospitals (Fig. 3). Concurrently, more than 64% of planned index admissions were to DPCs.

**Table 1:** Individual characteristics at index admission^a^

		Discharged from (index)				
	All	Unplanned			planned	
		DPC	Mix	Hospital	DPC	Hospital
	***N*** = 16,185	*n* = 2912	*n* = 1242	*n* = 5756	*n* = 4030	*n* = 2193
**Sex (%)**						
Female	**58.9**	61.4	59.1	52.2	62.4	67.2
**Age**						
Mean (SD)	43.0 (16.4)	42.7 (15.4)	43.2 (15.1)	41.8 (16.6)	43.5 (15.9)	45.6 (19.4)
Median	**42**	42	43	40	43	39
18–29	**25.2**	23.6	22.8	28.6	22.0	25.7
30–39	**19.8**	19.9	19.5	20.1	20.1	18.1
40–65	**44.9**	48.4	49.8	41.8	49.8	36.8
66+	**10.2**	8.1	8.0	9.5	8.1	19.4
**Diagnosis (%)**						
F20-F29	**25.6**	22.4	27.3	31.1	24.5	16.2
F30-F31	**13.6**	14.5	17.3	15.3	13.1	7.0
F32-F39	**27.7**	33.6	31.2	23.4	28.9	26.8
F40-F49	**22.9**	21.6	17.0	20.8	23.7	32.4
F50-F59	**2.7**	1.0	0.7	1.1	2.3	10.8
F60-F69	**7.5**	6.9	6.4	8.3	7.6	6.8
**Comorbid substance abuse (%)**						
Yes	**9.0**	6.4	8.9	12.5	5.9	8.6
**LOS days (%)**						
Mean (SD)	**34.8 (76.2)**	24.8 (48.9**)**	46.8 (51.6)	25.7 (58.6)	35.5 (70.9)	63.1 (135.6)
Median	**14**	11	31	8	20	25
2–7	**29.6**	30.8	7.5	45.0	13.5	30.5
8–14	**17.2**	24.3	12.5	16.8	19.3	7.6
15–30	**22.9**	23.6	27.9	16.0	34.7	15.5
31–60	**16.2**	12.6	29.6	12.2	20.3	15.6
61+	**14.2**	8.7	22.5	10.0	12.2	30.8
**Travel time DPC minutes (%)**						
Mean (SD)	**25.2 (31.0)**	20.9 (24.5)	24.3 (30.0)	25.1 (30.9)	28.2 (34.4)	26.4 (32.3)
Median	**11.6**	10.6	12.5	10.8	12.6	10.9
0–14	**61.7**	67.6	62.3	63.3	55.3	61.5
15–29	**13.9**	13.3	15.1	12.9	16.2	12.1
30–59	**14.6**	12.9	13.7	14.0	16.4	15.6
60–89	**5.0**	2.9	4.2	5.0	6.5	5.5
90+	**4.8**	3.4	4.8	4.8	5.7	5.3
**Travel time hospital minutes (%)**						
Mean (SD)	**57.5 (94.7)**	48.6 (76.3)	51.8 (78.4)	52.0 (93.8)	71.4 (107.8)	60.9 (99.0)
Median	**24.8**	20.4	24.9	20.4	35.8	24.2
0–14	**37.3**	39.4	35.2	43.3	26.3	40.3
15–29	**20.1**	22.6	21.6	20.1	19.7	16.9
30–59	**20.3**	19.4	20.5	18.1	24.2	19.8
60–89	**6.9**	5.8	8.0	5.9	9.0	6.8
90+	**15.4**	12.8	15.1	12.7	20.8	16.2
**Readmitted within 30 days (%)**						
Yes	**15.1**	16.7	18.4	17.9	11.2	10.5
**Readmitted within 365 days (%)**						
Yes	**47.7**	48.8	53.3	45.7	47.7	47.8

^a^There were only 52 discharges from Planned Mix index, due to their low numbers, these are omitted

Compared with all patients, patients discharged from an *unplanned DPC* index admission were more often middle aged (40–69 years old) women diagnosed with depression or other affective disorders (ICD-10 chapter F30) and had had an intermediate (8–30 days) LOS index hospitalization. In contrast, patients discharged from an *unplanned hospital* index admission were more often young (18–29 years old) men diagnosed with schizophrenia or psychotic disorders (ICD-10 chapter F20) or personality and behavior disorders (ICD-10 chapter F60), had comorbid substance abuse (Chapter F10) and had been admitted for short (2–7 days) LOS index hospitalizations. Those patients having had unplanned index admissions comprised of stay *both at DPC and hospital (mix)* more often had long (15+ days) index hospitalizations (Table 1).

Compared with all patients, patients discharged from a *planned DPC* index admission were more often middle aged (40–69 years old) women who had had an intermediate (8–60 days) LOS index hospitalization. In contrast, patients discharged from a *planned hospital* index admission were more often men, old (66+), were diagnosed with anxiety disorders (ICD-10 chapter F40) or behavioral syndromes (ICD-10 chapter F50) and had been admitted for long (60+ days) LOS index hospitalizations.

As can be seen in Fig. 3 left panel, study participants from rural regions of northern- or the mountainous regions of the south of Norway have average travel times to nearest psychiatric hospital of 90 min or more. Mean travel time to hospital was 57 min, with a maximum of 13.5 h.

Even with the much larger number of DPCs, study participants from several municipalities have average travel time exceeding 90 min (Fig. 3 right panel). Mean travel time to DPC was 25 min, with a maximum of 4.5 h.

As can be seen in Table 2, close to half of the then 422 Norwegian municipalities had an average travel time to hospital of more than 90 min. Only a few of the most densely populated municipalities had an average travel time to hospital of less than 15 min. Municipalities had more uniformly distributed average travel time to DPC.

**Table 2:** Number (%) municipalities by mean travel time in minutes to DPC and hospital

	0–14	15–29	30–59	60–89	90+
Distance to DPC – N (%)	81 (19.2)	71 (16.8)	112 (26.5)	69 (16.4)	89 (21.1)
Distance to hospital – N (%)	26 (6.2)	45 (10.1)	93 (22.0)	62 (14.7)	196 (46.4)

### Overall readmission

Out of the 16,185 individual patients included in the analyses, 15.1% were readmitted within 30 days, and 47.7% were readmitted within 365 days (Fig. 2).

**Fig. 4:**
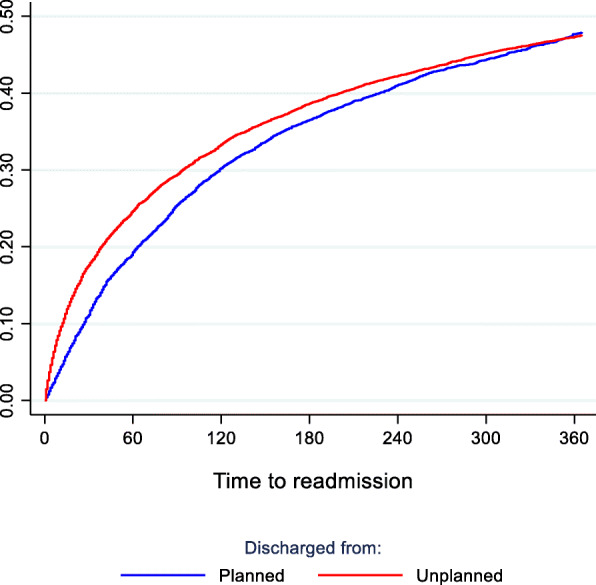
All psychiatric readmission within 365 days, by planned/unplanned index hospitalization

Patients discharged from unplanned index admissions were more likely to be readmitted within 30 days compared with those discharged from planned index admission, this difference could not be found after 365 days (Fig. 4).

**Fig. 5:**
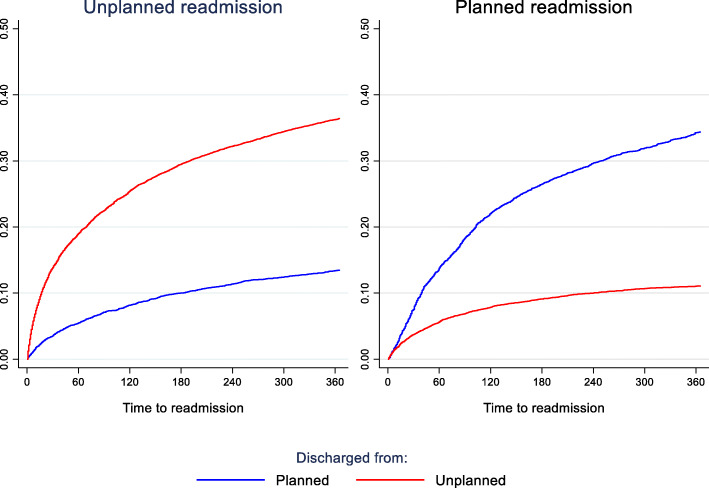
Unplanned and planned psychiatric readmission, by unplanned/ planned index hospitalization

Stratifying on unplanned vs. planned index admissions, we see that unplanned index admission more often leads to unplanned readmission while planned index admission more often leads to planned readmission (Fig. 5). Within 30 days, approximately 15% of unplanned index admissions had been unplanned readmitted while approximately 10% of planned index admissions had had an unplanned readmission. The long-term outcome is comparable, but the curve for unplanned readmission of unplanned index admissions is steeper in the early phase.

### System differences in readmission

**Fig. 6:**
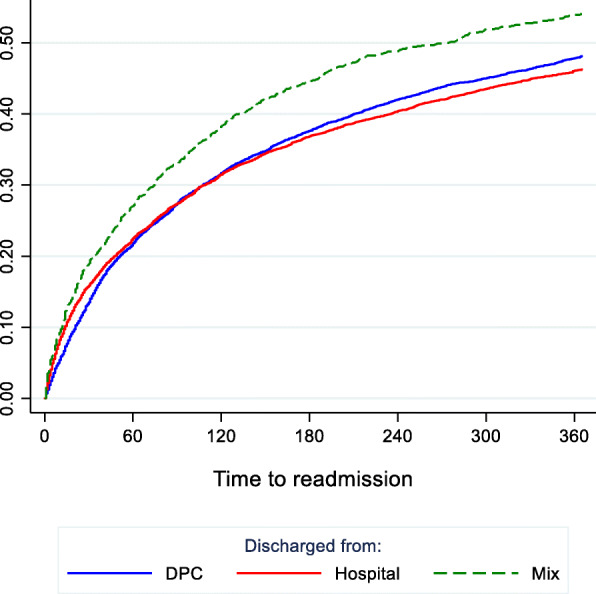
All psychiatric readmission within 365 days, by index discharge from DPC, hospital or mix

During the first 30 days, there was a significant difference in readmission rate between those having been admitted to hospital versus DPC during their index hospitalization, with a lower rate for DPC. However, after approximately 3 months these curves crossed, with a rate of readmission from DPCs further rising during the 365-day observational period, albeit not to a level significantly higher than for hospitals (Fig. 6). The readmission rate for those having had an index stay comprised of days both in hospital and DPC was higher throughout the 365-day period, and significantly so after about 40 days.

**Fig. 7:**
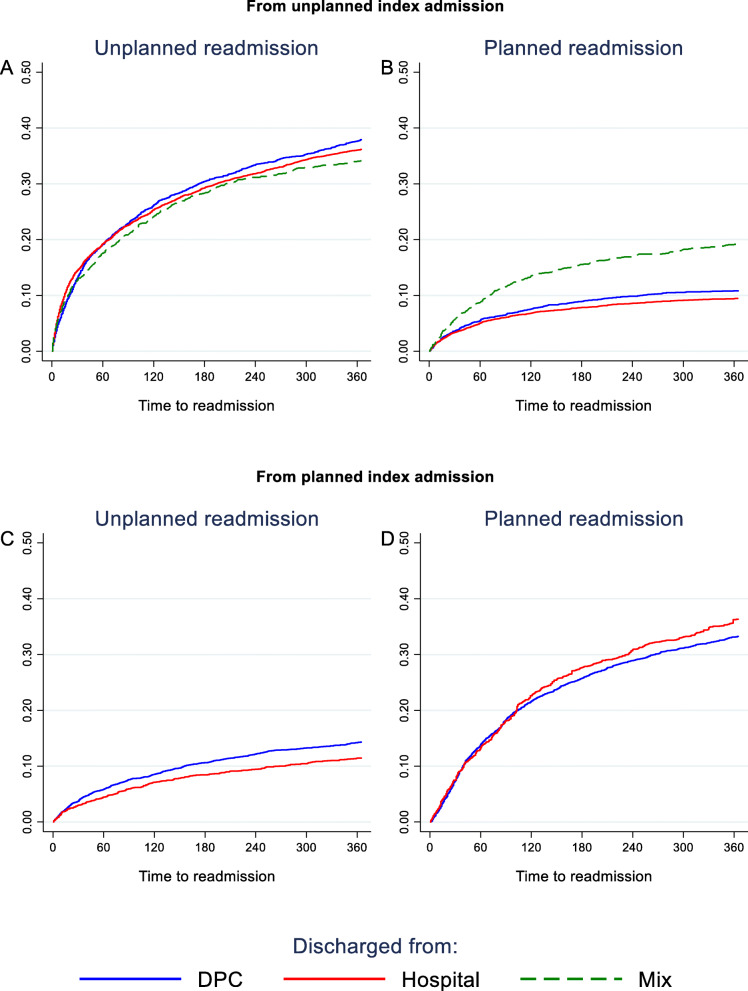
Unplanned and planned readmission from unplanned and planned index admission, by index discharge from DPC, Hospital or Mix. In panel **b**, Mix is significantly different from DPC and Hospital at 365 days. No other significant differences were found

Stratifying this finding by unplanned vs. planned index admission shows no differences between being discharged from hospital vs. being discharged from DPC (Fig. 7). However, for patients discharged from an unplanned index admission with a combination of treatment in both psychiatric hospital and DPC (i.e. mix), the risk of planned readmission was elevated.

**Fig. 8:**
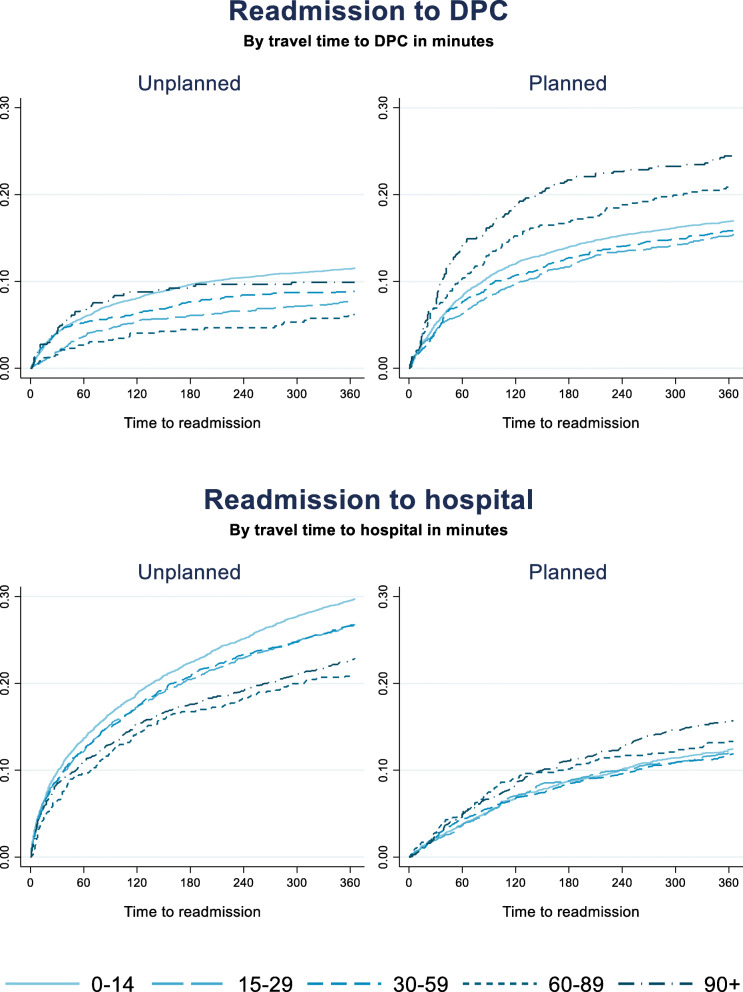
Readmission to DPC and hospital stratified by unplanned and planned readmission. By average travel time in minutes at municipality level

Average travel time to nearest DPC/hospital had different effects on readmission within 365 days depending on whether the readmission was unplanned or planned (Fig. 8). For unplanned readmissions to DPC, the rate was highest for those living closest to a DPC. The difference was significant at the 95% level compared to all groups but for those with travel time exceeding 90 min. The results indicate the same effect for unplanned readmissions to hospital. Here the group with travel time less than 15 min were significantly more often readmitted than all other groups.

For those having planned readmissions a travel time of more than 90 min is associated with a higher rate of readmission compared with all other groups. The pattern holds also for those readmitted to hospital, however with a lower level of statistical significance.

## Discussion

Although readmission rates long have been used as a quality indicator of inpatient health care, a full understanding of what they are comprised of and represent is limited. This study provides an examination of characteristics of patients admitted and readmission rates in a multi-level mental health care system as well as the effect of travel-time on readmission risk.

A main lesson learned from this study is that readmission rates must be interpreted in relation to local context. The structure of mental health care services in Norway reflects special geographical and demographic challenges. Mainland Norway spans 13 degrees of latitude, stretching over about 1750 km from South to North. Norway is the 6th largest country in Europe in terms of area but ranks 27th in terms of population. This implies that inhabitants in rural areas must travel far to go to hospital. Hence the need for mental health inpatient services closer to where people live in order to reduce travel times are clearly present. The development of the special community mental health centre model of DPCs with inpatient services meets this need. However, even though the number of DPCs are high and scattered around the country, the travel time to DPC is long in several of the less populated rural municipalities. This is likely to affect the system for treatment and follow-up of mental health patients.

Norway has a high volume of planned admissions in specialist mental health care, with a high share in DPCs. Our results suggest that 1 year after discharge, planned patients have the same likelihood of being readmitted as unplanned patients. Our results show that patients with long travel time to DPC or to hospital had a higher risk of planned readmission. This may indicate that readmissions are part of planned treatment courses when outpatient care is not easily accessible. Recent studies showing a negative effect of distance on outpatient mental health care utilisation supports such an assumption, e.g. [34–36]. Studying health care visits in general among rural dwellers, Arcury et al. [37], found distance to care to reduce utilisation of regular check-up visits, but not of acute care visits. They also found having a driver’s license to be positively associated with regular follow-ups and chronic care visits but having no association with acute care visits.

Our results suggests that distance to services may also affect utilisation of inpatient services in terms of readmission rates. We find that unplanned admissions within 1 year after discharge was more likely for those living in close proximity to a DPC or a hospital department. This may indicate that accessibility to some degree is driving readmissions and is in accordance with a common observation that utilisation rates are inversely related to distance to healthcare services [38], which is also found for admission to acute psychiatric hospitals in other countries [39–41] as well as outpatient, day-care and home-based services [14]. However, we do not find a consistent gradient between unplanned readmissions and distance to services. This in line with the results of Kalseth and Halvorsen [42] finding a complex relationship between travel time to hospital and dying in hospital in Norway.

The results also illustrate that readmission rates reflect task division between the different levels of mental health care services in Norway. The DPCs serve a local hospital function and provide more planned and more general psychiatric care, while the hospitals provide much of the unplanned activity and specialized mental health care. This is reflected in the descriptive statistics showing that there are significantly different groups of patients receiving psychiatric health care in hospital departments and DPCs. These groups have different readmission risks [10] and the difference in readmission risk between hospital departments and DPCs disappeared when we stratified index admissions as well as readmissions by urgency type (unplanned vs planned). That is, we found no indication that whether patients are treated at hospital or DPC impacts the risk or rate of readmission. This result also underscores the importance of addressing differences in specialization and functions when comparing readmission rates.

We find, however, that patient with an unplanned index episode spanning stays at both hospital department and DPC have a higher likelihood of having a planned readmission, i.e. being more likely to be followed-up by planned inpatient admissions. These patients typically have longer LOS than patients with unplanned episodes of care in either a hospital department or a DPC. More than 50% had LOS > 30 days in the mixed unplanned index group compared to about 20% in the hospital or DPC only unplanned index groups. This could indicate that they have more comprehensive care needs and that the extensive use of inpatient services reflects a more severe clinical condition than short inpatient stays because of life-circumstances and/or substance abuse.

As previously reported, overall 30- and 365-day readmission rate was found to be 15.1 and 47.7% respectively in this population [10]. This is considerably higher than what is found in other countries (ibid.) and what is reported from previous research, e.g. Tulloch et al. [6] reports 8 and 30% readmission at 30 and 365 days respectively among general psychiatric discharges. The current study provides additional insight on the high readmission rates in Norway by stratifying index admissions by provider type (hospital/DPC) and urgency type (unplanned/planned). As discussed, stratification by provider type is essential to capture task divisions. Stratification by urgency type is important, as the use of readmission rates as quality indicator refers typically to unplanned admissions and readmissions. However, mental health care systems may differ in the degree of planned activity it performs. Further, typically, comparison of readmission rates by use of register data often hampers proper exclusion of planned activity. Thirty-day rate of unplanned readmissions for severe mental illness is suggested as quality indicator for hospital care since if given adequate care and discharge planning, patients would not be readmitted within this short time after discharge. We find that the unplanned 30-day readmission rates for unplanned index patients are lower, varying from 13.1–14.5% depending on which provider type was involved in the index episode, than for the overall 30-day readmission rate (15.1%), but still on the high side compared to overall rates for many other European countries [10].

Longer term unplanned readmission rates (e.g. 1 year) are said to be indicators of lack of community follow-up and care continuity. Again, we find unplanned readmission rates for unplanned index patients to be lower, from 34 to 38% depending on provider type involved in the index episode, compared to overall 365-day readmission rate (47.7%). Hence, excluding planned activity has a larger effect on the 365-day readmission rate than the 30-day readmission rate. This relates to the observation that unplanned readmissions increase more than planned readmissions in the first days/months after discharge, but after 1 year the rates for unplanned and planned readmissions are comparable. This again, illustrates the importance of planned inpatient follow-up in the Norwegian mental health care system. Compared to many other European countries, Norway have high resource use in both specialized outpatient care and municipal mental health care [16, 43]. Hence, it does not seem that the high rate of planned admissions and readmissions observed, is because of low level of outpatient community care.

Lien [44] argues that patient hospitalization with following discharge represents a discontinuation of care. This is not necessarily the case. Myklebust et al. [45] found that “extensive decentralization of the psychiatric services positively affected coordination of inpatient and outpatient services”. Hence, for patients in DPCs, where the same staff often serves both in- and out-patients, discontinuation of care may not be a concern. In support of Myklebust et al. [45], Omer et al. [46] found in a systematic review that continuity of care systems, i.e. where the same clinicians are responsible for a patient’s care across inpatient and outpatient settings, gave better outcomes and stakeholder preferences. Likewise, planned inpatient follow-up may provide care continuity, especially for patients living far away from specialist services.

A previous Norwegian study found that patients having an unplanned (defined as urgent and involuntary admissions) compared to elective admission more often were suffering from severe mental illness and had low functional level, had higher risk of suicide attempt and of being violent (Ose et al. 2018). They also were less likely to have received outpatient treatment and more likely to have had prior unplanned admission during the 3 months prior to admission, compared to elective admissions. Furthermore, they were less likely to have had prior GP visits or using general home nursing or other municipal care services, but more likely of using municipal housing. This patient group is often difficult to treat and follow-up. Poor treatment engagement leading to worse clinical outcomes and symptom relapse can be a key determinant of high risk of acute readmissions [47]. Hence, treatment and follow-up approaches that contribute to higher level of engagement, such as person-centered and recovery-oriented care (ibid), may be effective for reducing unplanned readmission rates. Recovery orientation is the foundation of mental health rehabilitation programs. Inpatient rehabilitation services may be necessary for service users with very complex needs and severe symptomatology or psychosocial impairments, i.e. the patient group found to have unplanned admission to specialist care. Tsoutsoulis et al. [48] found nonacute inpatient mental health rehabilitation to reduce re-hospitalization compared to matched normative clients from community mental health services. We found that patients having stays comprised of days in both hospital and DPC are more likely to be planned readmitted.

### Limitations and strengths

A core strength of the current study is the ability to distinguish between planned and unplanned index admissions as well as readmissions. Another strength is the inclusion of an entire population as study participants. While previous studies often exclude planned (re)admissions [49], or only selected hospitals/providers within a health care system [12, 50]. By including all discharges from psychiatric inpatient care in Norway for a full year, and not focusing on a limited geographical area or a subset of index admissions, our results should be generalizable at least nationally and to other similarly organized health care systems.

Methodologically is the use of graphical analysis, by means of Kaplan-Maier failure plots, a strength. From our results we learn that factors significantly predicting readmission at 30 days may not at 365 days and vice versa. Focusing on rapid readmissions increases the chance of the readmission being connected with the previous hospitalization, yet for increased comparability between studies and from a systems perspective including longer intervals are valuable. The graphical representation of readmission rates over time provides the opportunity to see exactly what happens and when and if lines cross. Unfortunately including confidence intervals in the graphs would clutter them beyond readability.

The main limitation of the current study is that it relies solely on administrative data from one source, and hence does not comprise information on clinical conditions of patients or social circumstances, preceding and subsequent outpatient and GP care. Neither do we have knowledge regarding prior hospitalizations.

Given that the primary object of the present study was to be descriptive, we opted not to include controls for age, sex, diagnosis, LOS etc. because this likely would have eliminated or disguised the system differences.

## Conclusions

Geography, or travel time, appears to affect readmission rates. As do having had an index hospitalization comprised of days in both hospital and DPC during the same episode, which affects the risk of readmission initially but not in the long run. Our results suggest that readmission-rates in mental health services are influenced by multiple factors, and further studies should therefore incorporate both clinical variables of patients and service-characteristics as well as multi-level analyses. Travel time seems to be a variable for further research. Our results stress the importance of addressing differences in organisation and task distribution when comparing readmission rates between mental health systems.

## Data Availability

The analysis was based on register data. Data from the registries are available for research projects approved by the registries and that meets the requirements of the Health Research Act and the Personal Data Act, so the authors are not allowed to share the data. For data requests contact: service@helsedata.no.

## Abbreviations

DPC: District Psychiatric Center
GP: General physicians
ICD: International Classification of Disorders
LOS: Length of stay
NPR: Norwegian Patient Register
PNH: Psychiatric nursing homes
